# The Parson and the Public House
*"Village Public Houses." By a Proprietor, Rev. Osbert Mordaunt, Rector of Hampton Lucy, Warwickshire. (Warwick: H. T. Cooke and Sons.)


**Published:** 1893-02-18

**Authors:** 


### The Parson and the Public House.*

What should a conscientious clergyman do when, among other things, he inherits a public-house? A good many people would at once answer, "Shut it up." They don't think very carefully over the possible results of this shutting up, but have a feeling that it is not becoming for a parson to have anything to do with the profits of intoxicating liquor. Some few, indeed, who are believers in the need for total abstinence, object on principle to any one being permitted to sell drink—alas! that the good word drink should be degraded to signify only the materials for inebriety—and would encourage our parson to show the world the spectacle of a parish without a public-house within its bounds. These advisers we can honour; they may be fighting with windmills—things that have a usefulness of their own—but if they see in them devouring and devasting giants, they are right to do all they can for their overthrow. But the majority give their advice in simple obedience to a conventional idea of a clergyman's duty. He may take a glass of wine himself, and no one will blame him; but as he must reprove drunkenness, it is not discreet that he should, to public knowledge, have anything to do with the sale of that which makes drunk. To shut up the public-house, or even sell it to some one not hampered by the restrictions of "the cloth" is a way of getting out of the ungraceful position; but is it a solution of the difficulty?

A practical politician, be he a village Hampden or the autocrat of half a world, has to take things as they are and legislate accordingly. To make Utopian laws is to ensure their being broken—in the case in question, to have liquor smuggled into the village in ginger beer bottles, to have the grocer sell it and mark it in his books as tea, to have young men walk to the next village for muddy ale, and old ones bring bottles of spirits from the market town and sit and soak at home. More than forced abstinence is implied in the restriction; the feeling of liberty is aroused and becomes defiance, and the idea of outwitting the restrictions of a tyranny has a delight of its own. If it is certain that the villager will drink beer, a clergyman who thinks more of his parishioners than of the opinions of every conventional critic, will begin to ask himself if it is not better for them to buy good beer from himself than to get bad beer from some less responsible vendor.

This is the problem which came to the Rev. Osbert Mordaunt, and in a brief and practical pamphlet he has stated his experience as the proprietor of a village public-house. He decided to keep on the inn himself rather than risk a licence being obtained by someone over whom he could exercise no control, and he is not merely landlord of the house, but owner of the business. To satisfy those whose sense of propriety would be shocked by the notion of a clergyman making money out of such an establishment, it may be at once stated that he returns to the villagers in various charities the money he receives for refreshments. The profits of two years went to improve the water supply, so Mr. Mordaunt cannot be accused of trying to profit by the thirst of his parishioners; but the mode in which he deals with the profits is, after all, a detail, to be imitated or not, which is but a small part of the good that results from his ownership. The chief benefit is that his customers get good beer—beer which quenches thirst instead of increasing it, beer free from salt or tobacco, or subtler adulteration. The villagers are not poisoned with what they drink. This is, at least on the physical side, no small benefit. The cases of helpless drunkenness which we see both in town and country, when men come staggering out of a tavern, and after circling vaguely for a minute or two, fall down like poisoned rats—such cases are not due to the excessive consumption of any honestly-made intoxicant. These men have not often money enough to spend on drink to bring them to such a state; but the stuff sold to them is a poison whose immediate effect on brain and limbs is prophetic of the mischief it produces in the whole man in time.

But it may be asked how Mr. Mordaunt can be sure that the beer sold at his public-house, though pure when it comes from the brewery, is not tampered with before it reaches his customers. Does he stand behind the bar himself? No, he does not, though it would imply no discredit if he did. But the man who does stand there and dispense the beer is a salaried servant, who has no interest in the sale, no profit from it. He is forbidden to give liquor to any who seem to have had enough, and there is nothing to be gained by disobedience. The only result would be that he would lose a house which he occupies rent free, and would be deprived of the chance of earning the legitimate profits allowed him—those made by selling food in the place and letting carriages. The result is that in Hampton Lucy people still drink beer, but they don't get drunk.

Of course to Mr. Mordaunt, who maintains this inn from philanthropic motives—truly and practically philanthropic as those of any teetotaller—the question of profit from the establishment is not an important one. But it cannot be assumed that every one who keeps a public-house can afford to disregard it. Although he does not offer his example as an incitement to the average publican, who will probably always seek to grow rich by the quickest methods, however dubious, he does not expect the landlords and clergy to whom he does appeal to lose money by the undertaking. With the total receipts, a little over £300, he was able to spend in charity out of the profits as much as thirty pounds. The average tradesmen might think a tenth of the takings too small a return, but taking the matter as an ordinary investment it is an exceptionately large one Which of us would not be glad to get ten per cent. for our money? Therefore, any who incline to try this scheme, who, seeing that people will have fermented liquor in some form, are willing to give it them of a kind that shall do them no injury, need not be deterred by the dread of bankruptcy. The thing can be done without loss. It is for those to whom circumstances give a certain control over the doings of their fellows to decide whether in this unostentatious way they may not do a real good. They will be condemned by total abstainers; those who give their souls to mere convention will look askance at them; they will have much to bear in the way of misunderstanding and misrepresentation; but if they have courage to persevere they may make the public-house a kindly, harmless place instead of a curse to the country.

* "Village Public Houses." By a Proprietor, Rev. Osbert Mordaunt, Rector of Hampton Lucy, Warwickshire. (Warwick: H. T. Cooke and Sons.)

